# Effect of an educational program for the knowledge and quality of blood pressure recording^*^

**DOI:** 10.1590/1518-8345.3011.3179

**Published:** 2019-10-07

**Authors:** Ana Carolina Queiroz Godoy Daniel, Eugenia Velludo Veiga, Juliana Pereira Machado, Ana Carolina Cintra Nunes Mafra, Lyne Cloutier

**Affiliations:** 1 Hospital Israelita Albert Einstein, Unidade de Pronto Atendimento, São Paulo, SP, Brasil.; 2 Universidade de São Paulo, Escola de Enfermagem de Ribeirão Preto, Centro Colaborador da OPAS/OMS para o Desenvolvimento da Pesquisa em Enfermagem, Ribeirão Preto, SP, Brasil.; 3 Centro Universitário Barão de Mauá, Ribeirão Preto, SP, Brasil.; 4 Hospital Israelita Albert Einstein, São Paulo, SP, Brasil.; 5 Université du Québec à Trois-Rivières, Département de Sciences Infirmières, Trois-Rivières, QC, Canadá.

**Keywords:** Medical Records, Blood Pressure Determination, Education, Professional, Education, Continuing, Education, Nursing, Teaching Materials, Registros Médicos, Determinação da Pressão Arterial, Educação Profissionalizante, Educação Continuada, Educação em Enfermagem, Materiais de Ensino, Registros Médicos, Determinación de la Presión Sanguínea, Educación Profesional, Educación Continua, Educación en Enfermería, Materiales de Enseñanza

## Abstract

**Objective:**

to evaluate the effect of an educational program on blood pressure recording for nursing professionals in relation to theoretical knowledge and the quality of these records.

**Method:**

quasi-experimental study conducted in a hospital service located in the city of São Paulo. The theoretical knowledge of 101 professionals was measured using a validated questionnaire before and after the educational intervention; the quality of blood pressure records was evaluated using a validated form which was applied to 354 records in the pre-intervention period and 288 in the post-intervention period. The educational program was based on active teaching-learning methodologies and consisted of two strategies: expository/dialogue class and a board game. The Wilcoxon, Mann-Whitney, Fisher and Chi-Square tests were used for comparisons, adopting a level of significance of α=0.05.

**Results:**

the median of the professionals’ scores increased from 19 to 22 points in the post-intervention period (p<0.001). There was an improvement in the quality of the blood pressure recordings regarding the variables: cuff size (p<0.001), arm used in the procedure (p<0.001) and patient position (p<0.001).

**Conclusion:**

the educational program showed positive results in the promotion of knowledge among nursing professionals and in the improvement of the quality of blood pressure recording.

## Abstract (pt)

**Objetivo:**

avaliar o efeito de um programa educativo sobre o registro da pressão arterial para profissionais de enfermagem em relação ao conhecimento teórico e à qualidade desses registros.

**Método:**

estudo quase-experimental conduzido em um serviço hospitalar localizado no município de São Paulo. O conhecimento teórico de 101 profissionais foi mensurado por meio de questionário validado antes e após a intervenção educativa. A qualidade dos registros da pressão arterial foi avaliada utilizando-se formulário validado e aplicado a 354 prontuários no período pré-intervenção e 288 no período pós-intervenção. O programa educativo foi fundamentado em metodologias ativas de ensino-aprendizagem e constituído por duas estratégias: aula expositiva-dialogada e um jogo de tabuleiro. Os testes de *Wilcoxon*, *Mann-Whitney, Fisher* e Qui-Quadrado foram utilizados para comparações, adotando-se um nível de significância de α=0,05.

**Resultados:**

a mediana de acertos dos profissionais passou de 19 para 22 pontos no período pós-intervenção (p<0,001). Houve melhora na qualidade dos registros de pressão arterial quanto às variáveis tamanho do manguito (p<0,001), membro utilizado no procedimento (p<0,001) e posição do paciente (p<0,001).

**Conclusão:**

o programa educativo mostrou resultados positivos na promoção do conhecimento dos profissionais de enfermagem e na melhora da qualidade dos registros da pressão arterial.

## Abstract (es)

**Objetivo:**

evaluar el efecto de un programa educativo sobre el registro de la presión arterial para profesionales de enfermería en relación al conocimiento teórico y a la calidad de esos registros.

**Método:**

estudio cuasi-experimental conducido en un servicio hospitalario ubicado en la ciudad de São Paulo. El conocimiento teórico de 101 profesionales fue medido a través de un cuestionario validado antes y después de la intervención educativa; la calidad de los registros de la presión arterial fue evaluada utilizando formulario validado y aplicado a 354 prontuarios en el período pre-intervención y 288 en el período post-intervención. El programa educativo fue fundamentado en metodologías activas de enseñanza-aprendizaje y constituido por dos estrategias: clase expositiva-dialogada y un juego de tablero. Las pruebas de *Wilcoxon*, *Mann-Whitney*, *Fisher* y Qui-cuadrado se utilizaron para comparaciones, adoptando un nivel de significancia de α=0,05.

**Resultados:**

la mediana de aciertos de los profesionales pasó de 19 a 22 puntos en el período post-intervención (p<0,001). Se observó una mejoría en la calidad de los registros de presión arterial en cuanto a las variables: tamaño del manguito (p<0,001), miembro utilizado en el procedimiento (p<0,001) y posición del paciente (p<0,001).

**Conclusión:**

el programa educativo mostró resultados positivos en la promoción del conocimiento de los profesionales de enfermería y en la mejora de la calidad de los registros de la presión arterial.

## Introduction

Blood pressure (BP) recording is one of the steps of the indirect BP measurement. It provides important information for care and supports the clinical evaluation, the diagnosis and the therapeutic procedures used with patients affected by the most diverse health conditions^1-2^. In general, these records may enable the exchange of information between the members of the multi-professional team, the contact with the patient’s health history and the evaluation of the quality of care provided^3^.

National and international guidelines on hypertension (HTN) recommend that BP recording includes the position of the patient’s body, the arm used for cuff placement and the systolic blood pressure (SBP) and diastolic blood pressure (DBP) obtained immediately after performing the procedure^1-2^.

Despite the importance of BP recording for patient care, especially regarding diagnostic definition and planning of treatment and interventions, studies have shown that the conduct of nursing professionals regarding the BP documentation is still outdated and does not adequately portray the care received by the client^4-5^. In addition, the theoretical and practical knowledge about indirect BP measurement is below the expected for compliance with the technique^6-7^.

Educational interventions based on active teaching methodologies have been applied to nursing students and demonstrated positive results regarding the technique of indirect BP measurement^8-9^. However, the use of these strategies has been questioned due to the lack of theoretical foundation of the pedagogical method and the consequent impairment of the quality of evidences produced on professional training in health^10^.

In the organizational environment, ludic activities have been recognized for facilitating the construction of knowledge, improving the performance of health professionals in their daily activities and promoting adherence to the guidelines and protocols established in clinical practice^11^. The use of educational games in this context aims to facilitate the learning of concepts and techniques and to promote critical thinking, professional encouragement, social interaction, creativity, imagination, cognition and emotion^12^.

BP recording has been recognized as part of the indirect BP measurement for more than thirty years; however, its importance for the quality of care has been little discussed in the scientific and academic circles. In this sense, the development of research and the implementation of educational strategies regarding this documentation can support evidence-based practices and improve quality of care and patient safety in different levels of health care.

Given the above, the objective of the present study was to evaluate the effect of an educational program on BP recording for nursing professionals in relation to theoretical knowledge and the quality of these records. A secondary objective was to compare two educational approaches: expository/dialogue classes and expository/dialogue classes allied with the use of a board game.

## Method

This is a quasi-experimental study, conducted between December 2015 and June 2016, in a large emergency hospital in the city of Sao Paulo. Quasi-experimental studies are considered intervention studies and are different from true experiments because they do not include a randomization process or define a control group^13^.

The quasi-experimental before-and-after model with independent samples^14^ was used to evaluate the quality of BP recordings in the pre- and post-intervention periods. The before-and-after model with one group^14^ compared the theoretical knowledge of nursing professionals before and after the application of the educational program.

In order to detect the significant mean differences in the quality of BP recordings in the two periods, at least 88 observations would be necessary in each case, considering proportional comparison, effect sizes of 0.3, a significance level of 5% and 80% power^15^. The sample of medical records consisted of the total number of visits in the emergency unit, between the months of December 2015 and June 2016. Records from patients with emotional upheavals and/or psychomotor agitation, which could make it difficult to measure and periodically record BP were excluded. The physical and psychic conditions of these patients were identified through the records made by the medical and nursing staff during the period of care in this health service.

To compare the theoretical knowledge before and after the implementation of the educational program, all nursing professionals were invited to participate in the intervention, which was carried out between January and May 2016. The calculation of the sample size revealed that a minimum of 90 participants would be necessary to conduct a paired t-test to compare the scores obtained in the questionnaire applied to each professional, before and after the intervention period, considering a significance level of 5%, effect size of 0.3 and 80% power^15^. Considering that the intention of the researchers was to make the educational program available to all care staff, the sample consisted of the total number of eligible workers, which resulted in a larger number than planned.

Professionals who were away from work, those who refused to participate in the study, and those who did not complete all stages of the study were excluded. These stages were the signing of consent terms, completion of questionnaires and participation in educational interventions.

The quality of BP recordings was evaluated with a form called “Quality of Blood Pressure Recordings” (QBPR). The theoretical knowledge of nursing professionals was evaluated by a questionnaire called “Theoretical Knowledge on Blood Pressure Recording in Emergency Hospital Service” (TKBPR-EHS). Both instruments were developed in 2015 by the researcher of this study and validated in design and content by a group of six experts, who were chosen based on the following criteria: being a nurse, having a master or doctorate degree, having professional experience in the area of care, having methodological knowledge on the construction of questionnaires and scales and working in teaching or research with cardiovascular health, blood pressure measurement, hypertension, patient safety, educational innovation or related issues.

The QBPR was constructed based on literature documents on the measurement and recording of BP^1-2^. Its final version consisted of five items regarding records, namely: the measurement of the arm circumference (AC), the cuff size, the arm in which the AC was measured, the patient’s position and the BP values measured in millimeters of mercury (mmHg).

The TKBPR-EHS was developed based on a thorough review of the literature on nursing records and BP measurement^7,16-18^. It consists of six items related to the identification of the participant (date of birth, gender, professional category, time of professional training, time working in the health institution and work shift) and 23 items distributed in eight multiple-choice questions with four options of answer and three true or false questions composed of five sub-items. Overall, the TKBPR-EHS was composed of 11 questions that addressed the following themes: importance of BP recording for nursing care; BP recording in cases of hemodynamic instability; record of changed BP values; BP recording with automatic devices and its advantages; recording of patient position; recording of heart rate; recording of cuff size; BP recording according to hospital routine; frequency of BP recordings according to the patient’s clinical condition; frequency of BP recordings according to the nursing prescription; recording of the steps of the BP measurement procedure.

The TKBPR-EHS questionnaire was submitted to semantic validation by the DISABKIDS^®^ method^19^ in order to verify if all items of the data collection instrument were comprehensible to all members of the population to which it is intended. A convenience sample composed of three nursing professionals with different levels of education was chosen for this test, aiming to encompass all the different strata of the study population and to guarantee the reliability of the answers during the validation process.

The Delphi method^20^was used to systematize the opinions of experts regarding the judgment of the items of the QBPR form and the TKBPR-EHS questionnaire. The Content Validity Index (CVI)^21^ was applied to verify the degree of agreement between the specialists in each item of the instruments. The items that obtained CVI greater than or equal to 80% were considered valid.

The intervention was based on an educational program called “Pressure and Action”, developed by one of the authors of this study and based on documents prepared by the specialists in HTN^1,22-23^. The expository/dialogue class and the board game were the teaching strategies used during the intervention period. Both were based on the concept of active teaching-learning methodologies and on the perspective of autonomous education^24-25^.

For the expository/dialogue class, posters and illustrated badges with guidelines on the recording of the indirect BP measurement in a specific institutional form, with fields destined to the cuff size, the arm used in the procedure, the patient’s position, heart rate and BP values in mmHg were elaborated.

The board game consisted of 50 question-and-answer cards, five colored game pawns, a dice, an instruction manual and a board divided into five thematic axes: HTN physiopathology, BP concepts, BP oscillometric devices, BP cuffs, BP measurement procedure and BP recordings. For each thematic axis there were ten questions with their respective answers, each one illustrated in colored cards numbered from 1 to 10.

All phases of the data collection procedure were performed by one of the authors and conducted as follows:

Phase I (preliminary): the medical records cataloged in the Service of Medical and Statistical Archive were investigated in December 2015, before the implementation of the educational program, using the QBPR form.

Phase II (implementation): from January to May 2016, nursing professionals were recruited and invited to participate in the educational intervention, through an invitation letter that contained the Informed Consent Form (TCLE) and detailed information on the research, its objectives and its importance for their professional development. In a private room, the TKBPR-EHS questionnaire was delivered to each participant, immediately before and immediately after the intervention. A total of 38 professionals attended the expository/dialogue class and played the board game, and the remaining 63 only attended the expository/dialogue class, due to their workload and their unavailability to participate in the intervention. Groups of 38 and 63 professionals are sufficient to achieve an effect size of 0.6 when comparing the differences in performance between the participants^15^.

Phase III (exploratory): the medical records cataloged in the Service of Medical and Statistical Archive were investigated in June 2016, after the implementation of the educational program, using the QBPR form.

The effect of the educational program was evaluated by the Kirkpatrick Model^26^ in the learning and behavioral levels. The learning level was used to evaluate the theoretical knowledge of nursing professionals; the behavioral level evaluated the quality of BP recordings in the pre- and post-intervention periods.

The data collected were stored and typed twice in the program Microsoft Excel 2010. Then, they were processed in the program Statistical Package for the Social Sciences version 20.0 for Windows. The analyzes were conducted with the computer program R version 3.2.2. The pwr package was used for sample size estimates^15^

Descriptive analyzes were performed for all variables, which were described in absolute and relative frequencies, medians and quartiles. The Wilcoxon test for paired data was used to compare the performance of nursing professionals in the CTRPA-SHE. To compare the quality of BP recordings before and after the educational program, the Fisher’s exact test and the Chi-square tests were used for categorical measures and the Mann-Whitney test was used for numerical or ordinal measurements in independent samples. The Wilcoxon test for paired data and the Mann-Whitney test were used to compare the professionals who participated in the two strategies (classroom and game) and those who participated only in the expository/dialogue class. The significance level adopted for the tests was α = 0.05.

The study was submitted to the Research Ethics Committee of the mentioned hospital institution and was approved under protocol no. 31962014.1.0000.0071/2015.

## Results

**Figure 1:**
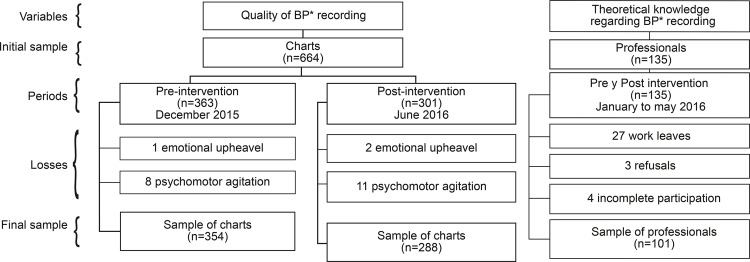
Schematic representation of the inclusion and exclusion of study participants according to the variables of interest and the intervention periods. São Paulo, SP, Brazil, 2015-2016

A total of 664 medical records were eligible, of which 363 were analyzed in the pre-intervention period and 301 in the post-intervention period. In the two periods, three medical records were excluded due to emotional upheavals and 19 due to psychomotor agitation (Figure 1).

*BP = blood pressure

The sample of professionals was composed of 135 participants, of whom 27 were excluded due to work leaves, three due to refusal to participate in the study and four because they did not complete all stages of the study. At the end, 642 medical records and 101 nursing professionals composed the respective samples (Figure 1).

Figure 1 shows the inclusion of study participant’s flowchart according to the variables of interest and the intervention periods.

The quality of BP recordings was analyzed in 354 records filled in the pre-intervention period (55.1%) and 288 in the post-intervention period (44.9%). In the pre-intervention period, two records (0.6%) contained AC measurement and another two (0.6%) had the record of the member in which AC was measured. In the same period, no records were found on the cuff size or on the patient’s position during the BP measurement procedure.

The analysis of the data on the post-intervention period showed an increase in the frequency of records with the variables “cuff size” (24.7%), “arm in which AC was measured” (29.2%) and “patient’s position” (69.1%). Table 1 describes and compares the quality of BP recordings in the charts analyzed in the pre- and post-intervention periods.

**Table 1:** – Description and comparison of the quality of blood pressure recordings identified in the charts analyzed in the pre- and post-intervention periods (n= 642). São Paulo, SP, Brazil, 2015-2016

Quality of the blood pressure recordings	Pre-intervention period (n=354) n (%)	Post-intervention period (n=288) n (%)	p-value*
Recording of AC measurement^†^	2 (0.6)	1 (0.3)	>0.999
Recording of cuff size	0 (0.0)	71 (24.7)	<0.001
Recording of arm in which AC was measured^†^	2 (0.6)	84 (29.2)	<0.001
Recording of patient position	0 (0.0)	199 (69.1)	<0.001

*Fisher’s Exact Test; ^†^AC = arm circumference

After the implementation of the educational program, there was a significant increase in the proportion of records containing the cuff size, the arm in which the AC was measured and the patient’s position (p<0.001).

The variable “BP recording with measurement in mmHg” showed compliance in 99.0% of the cases and did not show significant differences in the pre- and post-intervention periods (p= 0.900).

The sample of nursing workers was composed of 42 nurses, 58 nursing technicians and one nursing assistant. The characteristics of the professionals who participated in the study and were evaluated on their theoretical knowledge about BP recording before and after the educational program are presented in Table 2.

**Table 2:** Description of the professional characteristics of the participants of the educational program (n=101). São Paulo, SP, Brazil, 2016

Characteristics	No. (%)	Median [IQR]*
Age (years)		34.00 [29.00; 39.00]
Gender – Female	75 (74.3)	
Professional category		
Assistant	1 (1.0)	
Technician	58 (57.4)	
Nurse	42 (41.6)	
Time of professional training (years)		9.00 [4.92; 15.00]
Time working in the institution (years)		5.00 [2.66; 10.00]
Work shift		
Morning	37 (36.6)	
Afternoon	36 (35.6)	
Night	28 (27.7)	

*IQR = interquartile range

The frequency of correct answers in each CTRPA-SHE question was analyzed and is demonstrated in Table 3.

**Table 3:** Frequency of correct answers of nursing professionals in each question of the questionnaire “Theoretical Knowledge on Blood Pressure Recording in Emergency Hospital Service” in the pre-intervention and post-intervention periods (n=101). São Paulo, SP, Brazil, 2016

Questions	Pre-intervention (n)	Post-intervention (n)
1. What is the importance of BP* recording for nursing care?	82	93
2. Which BP* values recorded in the patient’s chart may be a sign of hemodynamic severity?	67	82
3. When verifying significant alterations in BP* values, one should:		
Ensure that the BP* measurement was performed with correct technique, otherwise it should be measured again.	101	101
Communicate the results obtained to the patient/companion.	82	91
Communicate the values obtained to the nurse/physician responsible for the patient.	101	101
Register in the nursing record that the nurse/physician was communicated, and the conduct was performed.	100	101
Communicate clinical engineering that there are errors in the device.	89	89
4. In relation to the advantages of using automatic devices for recording BP*, choose true or false:		
Accuracy of the results obtained.	79	95
Possibility of storing BP* values in the device.	90	99
Calculation of mean arterial pressure and values described in up to three digits.	95	97
Several measures can be performed in a time interval determined by the operator.	91	95
Rounding of values.	89	90
5. We should always record the patient’s position during BP* measurement, because:	96	97
6. We should always record the patient’s heart rate during BP* measurement, because:	46	79
7. To select and register, with practicality, the cuff size that best suits the patient’s arm, one should:	9	73
8. The BP* verification must be recorded:	99	100
9. To fill the nursing prescription item on the frequency of the verification of vital signs, the nurse should take into consideration:	99	99
10. In relation to the frequency of BP* recording, choose true or false:		
The BP* of unstable patients or patients receiving vasoactive drugs should be registered every 15 minutes at most.	94	101
The BP* of patients classified as high risk should be recorded every 2 hours at most.	78	100
The BP* of patients classified as very urgent should be recorded every 4 hours at most.	87	100
The BP* of patients classified as urgent should be recorded every 6 hours at most.	96	101
The BP* of patients classified as less urgent or nonurgent should not be recorded.	88	90
11. During and after the procedure of BP* measurement with automatic devices, the steps that should be recorded in the medical chart are:	63	96

*BP = Blood pressure

There was a significant improvement in the proportion of correct answers to question number 1 on “the importance of blood pressure recordings for nursing care” (p=0.006), question number 2 on “blood pressure values that may be a sign of hemodynamic severity” (p=0.001), alternatives a and b of question number 4 on the “accuracy of BP values obtained with the use of automatic devices” (p=0.001) and “possibility of storing BP values” (p=0.027), question number 6 on “heart rate recording” (p<0.001), number 7 on “cuff selection” (p<0.001), alternatives a, b and c of question number 10 on “frequency of BP recordings” (p=0.023, p<0.001 and p=0.002) and question number 11 on “recording of BP measurement steps” (p<0.001).

**Figure 2:**
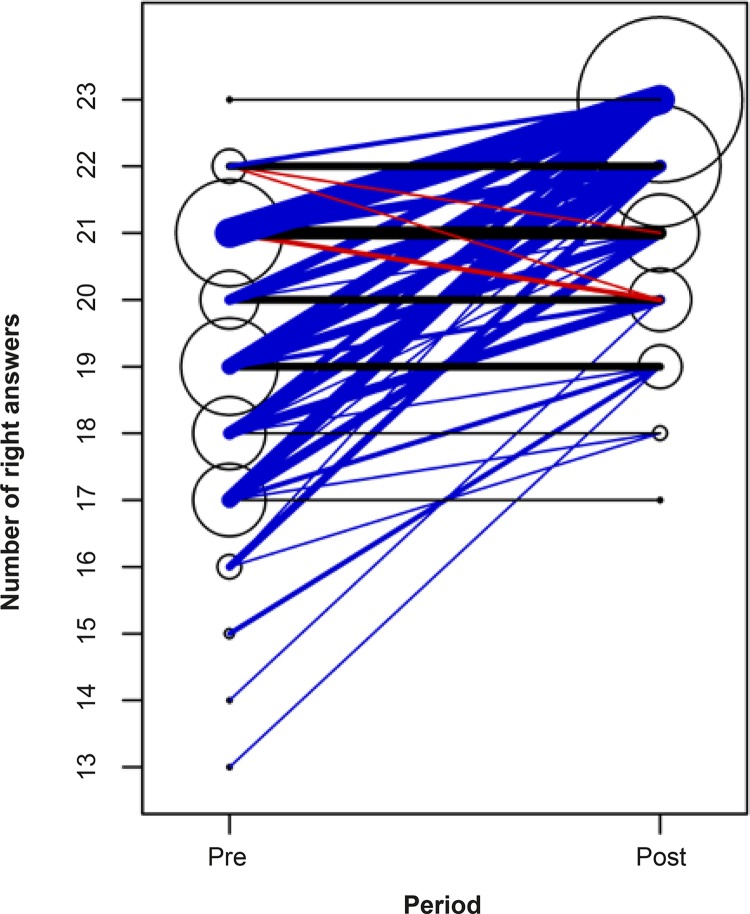
Total of right answers from nursing professionals in the questionnaire “Theoretical Knowledge on Blood Pressure Recording in Emergency Hospital Service” in the pre-intervention and post-intervention periods (n=101)*. São Paulo, SP, Brazil, 2016

Regarding the total score obtained in the CTRPA-SHE, 80 nursing professionals (79.2%) had a higher final score after the implementation of the educational program, 17 (16.8%) maintained the same score and four (4.0%) had lower scores. The median of the scores obtained in the pre-intervention period was 19 right answers (1st quartile 18 and 3rd quartile 21), while in the post-intervention period it was 22 (1st quartile 20 and 3rd quartile 23), out of a total of 23 items (p<0.001). Figure 2 shows the total number of right answers obtained by these professionals in the pre-intervention and post-intervention periods, when the CTRPA-SHE questionnaire was applied.

*Larger circles and thicker lines indicate a greater number of professionals. The evolution of each one is highlighted in different colors: blue if the score has increased, red if the score has decreased and black if it remained stable

The comparison between the professional categories regarding the number of right answers showed no significant differences in the performance of nursing assistants, technicians or nurses in the pre-intervention (p=0.759) and post-intervention (p=0.828) periods. The median score for all professional categories was 19 right answers in the pre-intervention period (1st quartile 18 and 3rd quartile 21) and 22 in the post-intervention period (1st quartile 20 and 3rd quartile 23).

A total of 63 nursing professionals (62.4%) participated in the expository/dialogue class, while 38 professionals (37.6%) participated in the class and in the board game. The median age of the participants in the board game was 31 years, and 84.2% were female. As for the professional category, 11 workers were nurses (28.9%), 26 were nursing technicians (68.4%) and 1 was a nursing assistant (2.6%).

The median of the scores obtained by the professionals who participated only in the expository/dialogue class was 19 right answers in the pre-intervention period (1st quartile 17 and 3rd quartile 21) and 22 in the postintervention period (1st quartile 20 and 3rd quartile 23). On the other hand, the median of the scores obtained by the professionals who participated in the expository/dialogue class and in the board game was 19 right answers in the pre-intervention period (1st quartile 18 and 3rd quartile 21) and 22 in the postintervention period (1st quartile 21 and 3rd quartile 22). The scores of these two groups did not show significant differences in the pre-intervention (p=0.412) and post-intervention (p=0.273) periods.

## Discussion

This study aimed to evaluate the effect of an educational program on BP recording for nursing professionals in relation to the theoretical knowledge and the quality of these records. Until the present moment, only few studies exploring this theme and its importance in the clinical and care context have been identified.

The indirect BP measurement comprises a series of steps that support the performance of the procedure in a correct and standardized manner, as specified by national and international guidelines on HTN^1-2^. The nursing record referring to the steps of this procedure is already clarified in the literature and consists of documentation of AC measurement, cuff size, arm used, patient position, time of measurement, values obtained without rounding, and heart rate^1-2^. The registration of all these steps is justified by the need to obtain reliable values that portray the clinical condition of the patient and contribute to the definition of a diagnosis and assertive treatment, monitoring and follow-up of health conditions.

The results showed a significant improvement in the quality of BP recordings when considering the variables documentation of the patient’s position, cuff size and arm selected for the measurement of pressure values, in the pre- and post-intervention periods. These results are in agreement with other studies in the literature, which used similar research methods and proved the effectiveness of educational strategies in promoting knowledge about the procedure of indirect BP measurement and its applicability in care practice^7-8^.

The choice of an appropriate cuff, based on the patient’s AC measurement, is necessary to obtain reliable BP values^27-28^. The cuff should correspond to 80% of the length and 40% of the width of the AC, in a length/width ratio of 2:1^2^. Cuffs of varying sizes are required to meet a range of arm circumferences; however, health professionals have been using a standard size for BP measurement, sometimes as an inadequate choice and sometimes due to unavailability of other cuffs or lack of standardization of measures by manufacturers^29^.

In recent years, alternatives to reduce misuse of the cuff have been studied and discussed by the societies of HTN specialists, with emphasis on the Canadian orientation which recommends the use of cuffs that delimit the AC interval and indicate the appropriate size through illustrated marks on the fabric surrounding the rubber bag^2^. Based on this new practice, this study demonstrated a significant improvement in the quality of the records referring to the size of the cuff selected. Guidance on the use of illustrated cuffs, as a substitute for AC measurement, can make the procedure more practical and contribute to the reduction of errors related to improper use of the equipment.

The choice of the patient’s arm is considered essential for individualized care, considering the need to identify the one with the highest blood pressure and make it a reference for the definition of the diagnosis^1-2^. The results regarding the record of the arm in which BP was measured were positive; however, less than 30% of the professionals who participated in the educational intervention performed the registration in practice, a fact that is similar to evidence found in previous studies^7^.

The registration of the arm used in the procedure indicates to other professionals possible situations that are contraindications for measurement in the contralateral limb, such as lymphedemas, thrombosis, grafting, ischemia, fistulas and the use of central or peripheral devices^27^. In addition, studies have demonstrated the existence of differences in SBP and DBP values in the different arms of older people and in patients with HTN, diabetes mellitus, hypercholesterolemia and peripheral vascular disease^30-31^.

The position of the patient during BP measurement is an essential information for the interpretation of the values obtained, definition of the diagnosis and decision of clinical procedures and nursing care. The SBP and DBP values present significant alterations when comparing sitting, lying and standing positions, due to the strong influence of hydrostatic and isometric factors on systemic vascular regulation^32^.

One of the most common error factors in the steps of indirect BP measurement is related to the rounding of BP values and the observer’s preference to register certain terminal digits, such as zero, two and four^33^. A study showed that the rounding of BP values can reach 70% in a given sample and it is directly related to the poor quality of care and lack of theoretical-practical knowledge to perform the procedure^7^.

The results of the present study showed that, even before the educational program, nursing professionals performed 99% of BP recordings with units of measurement compatible with the determination of the device, in mmHg. This may be associated with the use of digital oscillometric devices, which record BP values with up to three digits and minimize the observer’s preference to certain terminal digits.

Regarding the theoretical knowledge, the results showed higher scores in the CTRPA-SHE after the educational program, mostly on the questions on BP recordings in patients with hemodynamic instability, advantages of the use of oscillometric devices on BP recordings, the frequency of BP recordings according to the patient’s clinical condition and the recording of the steps of the indirect measurement of BP. These evidences demonstrate the positive effect of the intervention implemented in this study and the applicability of active teaching-learning methodologies in the context of nursing care.

The active methodologies use questioning as a facilitator of the teaching-learning process, since they lead the student to relate discoveries of phenomena of interest with their previous knowledge and with real-world experiences^34-35^. As teaching strategies based on this methodology, we emphasize expository/dialogue classes, group discussions, individual instruction, games, simulation and role playing, which promote the active participation of the student, the use of cognitive, affective and psychomotor skills and the development of theoretical-practical knowledge, critical thinking, clinical reasoning and problem solving skills^9,35-37^.

The use of innovative strategies has demonstrated effective results in improving theoretical and practical knowledge about the indirect BP measurement^7,9^. However, there are several evidences on the lack of quality of health records and on the existence of significant gaps in the knowledge of nursing professionals regarding documentation of patient care and practical application of nursing process steps^38-39^.

Nursing records and the indirect BP measurement are themes that are constantly addressed in nursing undergraduate courses, since they are part of the daily life of the health team and can help care planning. Despite this, the skills acquired in university education have not been sufficient to develop the knowledge and skills required by the labor market, which makes the educational role of health organizations a key part of professional training^40^. The same logic can be applied for technical courses for workers in that profession.

BP recording is considered a simple and easy task; however, the promotion of knowledge through the update of programs and continuing health education contributes to the technical and scientific training of professionals and to patient safety and quality of nursing care.

This study collaborated with the development of an innovative educational program, which was evaluated through the application of validated data collection instruments capable of measuring the theoretical knowledge of nursing professionals about BP recordings and investigating the quality of these records in medical charts of patients admitted to an emergency hospital service. The strategies demonstrated here can be adapted and applied in other health services for the promotion of health education.

There are some limitations in relation to the internal and external validity of the results presented in this study, such as the choice of non-probabilistic samples and the peculiarities of the site and the population selected. Experimental studies with more representative samples and with the implementation of active teaching-learning methodologies in other care contexts and in health care services with different levels of technological and financial investment should be carried out.

## Conclusion

The implementation of an educational program on BP recordings for nursing professionals showed positive results regarding the promotion of theoretical knowledge and the quality of BP recordings in a hospital service. The implementation of active teaching-learning methodologies contributes to the development of technical skills, the establishment of safe practices and the promotion of professional knowledge.
